# Novel Pathway for Corrinoid Compounds Production in *Lactobacillus*

**DOI:** 10.3389/fmicb.2018.02256

**Published:** 2018-09-25

**Authors:** Andrea Carolina Torres, Verónica Vannini, Graciela Font, Lucila Saavedra, María Pía Taranto

**Affiliations:** Centro de Referencia para Lactobacilos (CERELA)-CONICET, San Miguel de Tucumán, Argentina

**Keywords:** lactic acid bacteria, *Lactobacillus*, corrinoid synthesis, cobalamin gene cluster, biosynthetic intermediaries

## Abstract

Vitamin B_12_ or cobalamin is an essential metabolite for humans, which makes it an interesting compound for many research groups that focus in different producer-strains synthesis pathways. In this work, we report the influence of key intermediaries for cobalamin synthesis added to the culture medium in two *Lactobacillus* (*L.*) strains, *L. reuteri* CRL 1098 and *L. coryniformis* CRL 1001. Here, we report that addition of Co^2+^ and 5,6-dimethylbenzimidazole increased the corrinoid compounds production in both strains while addition of L-threonine increased only the corrinoid compounds production by CRL 1001 strain. Then, we purified and characterized by LC-MS the corrinoid compounds obtained. Physiological studies besides *in silico* analysis revealed that *L. reuteri* CRL 1098 and *L. coryniformis* CRL 1001 follow different pathways for the last steps of the corrinoid compounds synthesis.

## Introduction

Vitamin B_12_ belongs to group B vitamins and is the most complex water-soluble vitamin. Many analogous of vitamin B_12_ have been described; all of them are porphyrin compounds with a common structure: a corrinoid ring contracted with a chelated cobalt ion at the center of the macrocycle. In this vitamin, the cobalt ion is covalently bound to an upper or β-ligand, and is coordinated with a lower or α-ligand (Rucker et al., 2001). In nature, the vitamin B_12_ analogs present either an adenosyl group or a methyl group as β-ligand. The best-studied vitamin B_12_ is cobalamin, a cobamide where 5,6-dimethylbenzimidazole (DMB) is the α-ligand aglycon (Johnson and Escalante-Semerena, 1992). In the vitamin B_12_ synthetic preparations, the cyano group is present as β-ligand in contrast to naturally occurring analogs. Furthermore, changes in the α-ligand result in different vitamin B_12_ analogs. Several vitamin B_12_ analogs containing benzimidazoles, purines, and phenolic compounds as bases of the α-ligand have been described (Chan et al., 2015).

*De novo* biosynthesis of all these corrinoid compounds can be divided into three main steps: (i) the Uroporphyrinogen III synthesis; (ii) the corrinoid ring synthesis, and (iii) the adenosylation, the amino-propanol arm attachment and the nucleotide loop bridging assembly of the lower ligand to the cobalt of the corrinoid ring core (Martens et al., 2002). Corrinoid ring synthesis follows two different pathways according to oxygen requirements. In the aerobic pathway, the cobalt chelation occurs as one of the final steps of the corrinoid ring formation while in the anaerobic pathway this reaction takes place in the first step of the synthesis (Warren et al., 2002).

Animals cannot synthesize vitamin B_12_, which is involved in many important enzymatic reactions, thus making it an essential metabolite. These reactions are the conversion of homocysteine to methionine and the interconversion of (2R)-methylmalonyl-CoA to succinyl-CoA. Certain bacteria strains and archaea are able to synthetize vitamin B_12_ by *de novo* biosynthetic pathway (Roth et al., 1996). For this reason, many research studies are focused on the production of vitamins by bacteria in food and on improving the synthesis in large-scale production as well.

Currently, there are several *Lactobacillus* (*L*.) strains described as vitamin B_12_ producers, such as *L. reuteri* CRL 1098, *L. rossiae* DSM 15814, *L. coryniformis* CRL 1001 and *L. plantarum* BCF 20, BHM 10 and LZ 95 (Taranto et al., 2003; De Angelis et al., 2014; Torres et al., 2016; Bhushan et al., 2017) whose genomes have been partially sequenced.

Previous genome analysis of *L. reuteri* CRL 1098 and *L. coryniformis* CRL 1001 showed that both strains contain all necessary genes for *de novo* corrinoid compound biosynthesis. In this work, we put in evidence key differences in the last steps of the biosynthetic pathway of these strains. Besides, an increased corrinoid compounds synthesis was observed when adding certain intermediaries to the culture medium. These results were confirmed by HPLC quantification, and compounds were characterized by mass spectrometry. The expression profile of key genes of the biosynthetic pathway was also studied. Finally, we demonstrated the existence of two different biosynthetic pathways of cobalamin-type corrinoid compounds in *Lactobacillus* strains.

## Materials and Methods

### Strains, Media, and Culture Conditions

*Lactobacillus reuteri* CRL 1098 and *L. coryniformis* CRL 1001 were previously described as cobalamin producer strains (Taranto et al., 2003; Torres et al., 2016). These strains belong to CERELA-CONICET culture collection. The strains CRL 1098 and CRL 1001 were grown overnight without shaking in Man-Rogosa-Sharpe (MRS) broth and in Vitamin B_12_ Assay Medium (Merck, Germany), at 37°C.

*Salmonella (S.)* Typhimurium AR 2680 was used as indicator strain in the bioassays for cobalamin determination in minimal A medium (NaCl, 0.5 g/l, Na_2_HPO_4_, 6 g/l; KH_2_PO_4_, 3 g/l, NH_4_Cl 1 g/l; glucose, 4 g/l; MgSO_4_, 2 mM, CaCl_2_ 0.1 mM). This strain has two mutations in *metE* and *cbiB* genes. The AR 2680 strain was grown with aeration in Luria-Bertani (LB) broth at 37°C. As negative control, *L. plantarum* ATCC 8014 strain was used.

### Cultures and Cell-Extracts

The biosynthetic intermediaries added to the Vitamin B_12_ Assay Medium were: 5-aminolevulinic acid [ALA; final concentration (FC): 25 ng/ml]; Cobalt Chloride (CoCl_2_, FC: 250 ng/ml), 5,6-dimethylbenzimidazole (DMB; FC: 200 ng/ml); Porphobilinogen (PBG, FC: 250 ng/ml); L-threonine (L-Thr, FC: 50 μg/ml) and Uroporphyrinogen III (UIII, FC: 250 ng/ml). The corrinoids produced in the presence of different intermediaries were extracted as described by Torres et al. (2016).

### Cobalamin Detection

The production of corrinoid compounds in the cell extract (CE) obtained from each strain grown for 16 h was analyzed with the bioassay using *S.* Typhimurium AR 2680 as indicator strain. *L. plantarum* ATCC 8014 and a commercial cyanocobalamin solution (0.5 μg/ml) were used as negative and positive control, respectively. In addition, the concentration of vitamin B_12_ was determined by commercial enzyme immunoassay (RIDASCREEN-FAST Vitamin B_12_. R-Biopharm, Rhone Ltd., Glasgow, Scotland). The competitive immunoassay for the vitamin B_12_ determination was performed following the protocol described by the manufacturer.

### Purification, Characterization, and Quantification of the Corrinoid Produced by *Lactobacillus* Strains

Based on previous results of our research group, key intermediaries (alone or in combination) were added to the growth culture media in order to increase the corrinoid concentration produced. The following conditions were used for *L. coryniformis* CRL 1001 and *L. reuteri* CRL 1098: (i) CoCl_2_, (ii) CoCl_2_ plus DMB, and (iii) CoCl_2_ plus L-Thr only for CRL 1001 strain.

Then, the cells were broken and the corrinoid in the intracellular fraction (cell extract -CE-) was evinced by bioassay. The corrinoid produced was purified and quantified by RP-HPLC. The corrinoid concentration was calculated from the peaks areas and a commercial CN-Cbl standard curve made with 0.5, 1, and 2 μg/ml. The peaks of the chromatogram were collected for the mass spectrometry analysis. RP-HPLC and mass spectrometry were carried out as described by Torres et al. (2016). Experiments were performed three times in duplicate and results were expressed as means ± SD. Statistical analysis was conducted using MINITAB software (version 15 for Windows). Tukey’s *post hoc* test was used to test for differences between the mean values. Significance was set at *P* < 0.05.

### Relative Expression of Key Genes of Cobalamin Synthesis in *Lactobacillus* Strains

The primers for relative gene expression analysis (RT-qPCR) were designed on the basis of the corresponding gene sequences of *L. coryniformis* CRL 1001 (NZ_LNUL00000000.1) and *L. reuteri* CRL 1098 (NZ_LYWI00000000.1) using Primer Design tool (Bio-Rad) (**Table 1**). As normalizing reporter, *16S rRNA* gene was used.

**Table 1:** Primer sequences of key genes of *de novo* biosynthesis of cobalamin-type corrinoid compounds.

Strain	Gene	Forward primer (5′–3′)	Reverse primer (5′–3′)	Amplicon length (bp)
CRL 1098	*16S rRNA*	ACGTGCTACAATGGACGGTA	ACTAGCGATTCCGACTTCGT	120
	*cbiF*	TGCGTTCCTGGTGTTAGTTC	GTCCTGCCATACGCGTAATAA	106
	*hemB*	CGCGAAGTAGCTAGTGATGAA	CAACTAACGGCAGCAAAGTATG	115
	*cobS*	AAGCACATGGCGAATTATTG	TTCGTGACCTTTCGTCGAT	132
	*cobT*	AAACAGCAGCAGAAGTTGTTGGGC	CATTGCTCCAGCCATTGCACCTAA	182
CRL 1001	*16S rRNA*	GACGAAAGTCTGATGGAGCA	TTCTGGTTGGATACCGTCAA	122
	*cbiF*	AGTGTTTCTGGGACTTTGGC	CGGTATTCATGGGTGAAATG	146
	*hemB*	GATGGTATCGTGCAACAAGC	ACGACACCACAATGACCAGT	113
	*cobS*	TCTTCACGAACACCCGATAA	TTGCAATGGTGTTACCTGCT	141
	*pduX*	GTACGGCAGATCTAGTGGCA	GCATCGATCACAGTCAATCC	148
	*cblS*	TCAGTCGGTGTAACCGTGAT	GTCGCACTTCTTGGTAAGCA	148
	*cblT*	CAATTATGCATCAAGCCGTT	TGGTAAGGCCACGTAGACAA	98

Total RNA from *L. coryniformis* CRL 1001 and *L. reuteri* CRL 1098 grown in presence of the intermediaries were extracted at different growth phases (lag, exponential, and late exponential). Briefly, 10 mL of the cell pellet was suspended in 500 μL TE buffer (Tris–HCl 10 mM pH 8 – EDTA 1 nM pH 8) and added to 170 μL macaloid 2%, 500 μL TE buffer saturated with phenol-chloroform-isoamylic solution (chloroform-isoamylic 24:1, phenol-chloroform-isoamylic 1:1), 50 μL SDS 10% and 0,6 g glass beads (Sigma-Aldrich, Buenos Aires, Argentina). This suspension was subjected to Mini-BeadBeater-8 cell disrupter (BioSpec Products Inc.) at maximum speed in 10 cycles of 1 min each cycle, with intervals of 1 min on ice, to disrupt the cells. Subsequently, samples were centrifuged (5,000 ×*g*, 15 min, 4°C). The upper phase was separated and added phenol-chloroform-isoamylic solution (chloroform-isoamylic 24:1, phenol-chloroform-isoamylic 1:1). Again, the samples were centrifuged (5,000 ×*g*, 15 min, 4°C). After separating the upper phase, RNA precipitation was done with absolute ethanol and 3 M sodium acetate at -70°C overnight. Next day, samples were centrifuged (10,000 ×*g*, 15 min, 4°C), washed with ethanol 70°, and dried at room temperature. RNA samples were suspended in 20 μL miliQ water. DNase treatment was performed adding 10 μL of DNase (Turbo Dnase, Ambion, Thermo Fisher Scientific, Buenos Aires, Argentina) followed by incubation at 37°C for 180 min. DNase was inactivated by adding 1 μl of RQ1 DNase Stop Solution (Ambion) to the reaction mixture and heating at 65°C for 10 min. After verifying the absence of DNA by conventional PCR using the purified RNA as template, the samples were quantified with a Qubit^®^2.0 fluorometer (Invitrogen^TM^, Life Technologies Co., Carlsbad, CA, United States) using Qubit^®^HS RNA Assay Kit (Molecular Probes^TM^, Life Technologies Co.). All RNA samples were stored at -70°C until use.

The cDNA was synthetized using 1 μg of total RNA and qScript^TM^ cDNA SuperMix kit (Quanta Biosciences^TM^) according to the manufacturer’s instructions in a T100^TM^ Thermal Cycler (Bio-Rad). A conventional PCR was performed to confirm cDNA synthesis. The cDNA was stored at -70°C until use.

Quantitative PCR assays was performed using an iQ^TM^5 Multicolor Real-Time PCR Detection System iCycler (Bio-Rad Laboratories Inc.). Amplicons were detected with PerfeCta^TM^ SYBER^®^ Green SuperMix for iQ^TM^ (Quanta Biosciences^TM^). Each reaction contained 1X SYBER^®^ Green SuperMix, 300 nM of each primer and 50 ng of total cDNA, genomic DNA as positive control and no template as negative control. All reactions were done in duplicate. The amplification program consisted of 1 cycle of 94°C for 5 min and 40 cycles of amplification (94°C for 1 min, 55°C for 1 min, and 72°C for 30 s) followed by a melting curve (81 cycles of 10 s at 60°C). The relative expression of the *cbiF*, *hemB*, *cobS*, *pduX*, *cblS*, and *cblT* genes for *L. coryniformis* CRL 1001 and *cbiF*, *hemB*, *cobS*, and *cobT* for *L. reuteri* CRL 1098 with the addition of different synthesis intermediaries at 3, 6, and 9 h was estimated according to the 2^-ΔΔCT^ method (Livak and Schmittgen, 2001). The condition with the addition of CoCl_2_ at 3, 6, and 9 h was used as control for each point of the growth curve for each condition. The reported values are the changes in the gene expression of the strain grown with the intermediaries and without (control, given value = 1) and normalized against *16S rRNA* gene expression.

## Results

### *In silico* Comparative Genomic Analysis of Cobalamin Biosynthetic Gene Cluster

**FIGURE 1:**
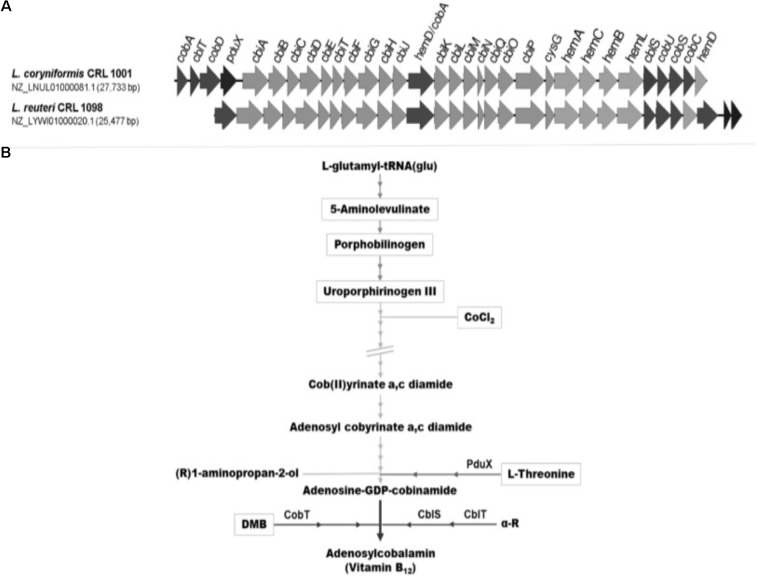
**(A)** *cbi*-*cob*-*hem* gene clusters of *L. coryniformis* CRL 1001 and *L. reuteri* CRL 1098. **(B)** Simplified cobalamin anaerobic synthesis pathway. Addition of different pathway intermediaries are highlighted with a box.

*Lactobacillus reuteri* CRL 1098 and *L. coryniformis* CRL 1001 are well-known cobalamin producer strains and they have gained attention for novel functional foods development. Previous *in silico* analyses of genome sequences of both strains revealed the presence of all the necessary genes for the *de novo* synthesis of cobalamin-type compounds although some differences in the cobalamin biosynthetic gene cluster were evidenced (**Figure 1A**). The genes *hemALBCD* encoding proteins involved in the first steps, the synthesis of uroporphyrinogen III from L-glutamyl-tRNA(glu), are present in both genomes. In addition, both strains produce the corrinoid ring by the anaerobic pathway and they bear all the *cbi* genes necessary for the complete ring formation. However, the last biosynthetic steps showed genes involved in different pathways for the aminopropanol arm formation and the assembly of the lower ligand.

**FIGURE 2:**
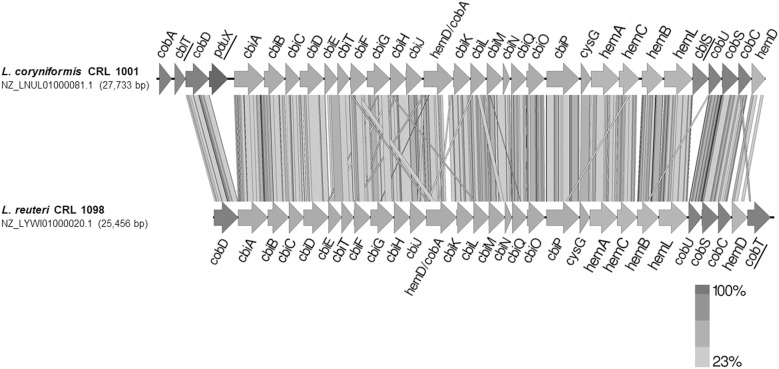
*cbi*-*cob*-*hem* gene clusters comparison between *L. coryniformis* CRL 1001 and *L. reuteri* CRL 1098. Arrows indicate the transcription direction depict genes. Orthologous conserved genes are depicted as gray and black bars.

The genome of *L. reuteri* CRL 1098 possesses *cobT* gene encoding for a nicotinate mononucleotide (NaMN): base phosphoribosyltransferase, which activates the lower ligand base. In contrast, *L. coryniformis* CRL 1001 does not own *cobT* gene but possesses the *cblTS* genes encoding for an α-ribasol transporter and a kinase protein, respectively, as described for other *Firmicutes* strains. The α-ribazole salvaging and α-ribazole-P synthesis were reported previously and presented as an alternative pathway of lower ligand activation (Gray and Escalante-Semerena, 2010). It is important to note that only *L. coryniformis* CRL 1001 harbors the *pduX* gene that encodes a kinase able to phosphorylate L-Thr. This compound is a precursor of the aminopropanol arm that binds the lower ligand to the corrinoid (**Figure 2**). The *in silico* studies point out differences in the set of genes involved in the cobalamin-type compounds synthesis. On this basis, the production of these compounds by the strains under study in the presence of different intermediaries was analyzed.

### Effect of Biosynthetic Pathway Intermediaries on Corrinoid Compounds Production

**FIGURE 3:**
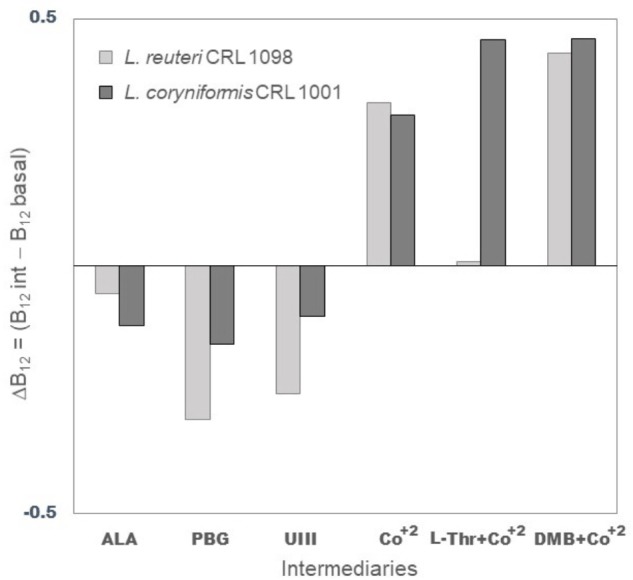
Relative quantification of corrinoid compounds produced by *L. reuteri* CRL 1098 and *L. coryniformis* CRL 1001 with addition of different intermediaries. Results are presented as ΔB_12_ (net difference of the corrinoid quantification with [B12 int.] and without intermediary [B_12_basal]). ALA (5-aminolevulinic acid), PBG (Porphobilinogen), UIII (Uroporphyrinogen III), CoCl_2_ (Cobalt, Co^+^), L-Thr (L-threonine) + CoCl_2_, DMB (5,6-dimethylbenzimidazole) + CoCl_2_.

*Lactobacillus coryniformis* CRL 1001 and *L. reuteri* CRL 1098 were grown in vitamin B_12_-free culture medium in the presence of the following biosynthetic pathway intermediaries: ALA, PBG, UIII, CoCl_2_, DMB plus CoCl_2_, L-Thr plus CoCl_2_. The sequential addition and roles of intermediaries have been shown in **Figure 1B**. The effect of DMB and L-Thr was evaluated in the presence of supplementary CoCl_2_ considering that the addition of this ion is an essential condition for the synthesis of corrinoids in stages subsequent to the insertion of this element. The **Figure 3** show the relative quantification using a commercial enzyme immunoassay of total corrinoids in the presence of different intermediaries respect to the condition without intermediaries. The corrinoid production by *L. coryniformis* CRL 1001 was greater with the CoCl_2_, DMB + CoCl_2_ and L-Thr + CoCl_2_ compared with the condition without intermediaries; ALA, PBG, and UIII did not improve the corrinoid synthesis in this strain. In *L. reuteri* CRL 1098, only DMB + CoCl_2_ and CoCl_2_ increased vitamin B_12_ production while the other intermediaries evaluated (L-Thr, ALA, PBG, and UIII) had no positive effect.

### Purification, Quantification, and Characterization of the Corrinoid Compounds Produced by *Lactobacillus* Strains

**FIGURE 4:**
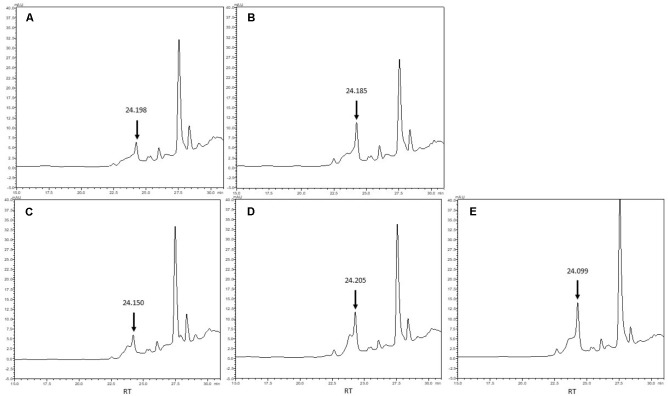
RP-HPLC chromatogram of the cell extract (CE) from *L. reuteri* CRL 1098 (**A** CoCl_2_ **B** DMB + CoCl_2_) and *L. coryniformis* CRL 1001 (**C**- CoCl_2_ **D**- DMB + CoCl_2_ **E**- L-Thr + CoCl_2_). An arrow indicates peaks corresponding to the corrinoid compound.

Bioassay and immunoassay demonstrated that both CoCl_2_ and DMB + CoCl_2_ addition increased the production of corrinoid compound by *L. coryniformis* CRL 1001 and *L. reuteri* CRL 1098; while the presence of L-Thr + CoCl_2_ induced the corrinoid production only in CRL 1001 strain. L-Thr was not evaluated in further assays for CRL 1098 strain as it had no effect on the corrinoid formation. To evaluate the optimum cobalamin production of CRL 1001 strain, a set of different growth conditions were evaluated (i) CoCl_2_, (ii) CoCl_2_ + DMB, and (iii) CoCl_2_ + L-Thr. In subsequent experiments, the corrinoid production by *L. reuteri* CRL 1098 was evaluated only in the presence of CoCl_2_ and CoCl_2_ + DMB. In order to purify the corrinoid compounds, the peaks with retention time (RT) close to cyanocobalamin (CN-Cbl) RT (24.98 min) were collected (**Figure 4**) and analyzed for cobalamin activity by bioassay using *S.* Typhimurium AR 2680 as indicator strain. All the collected peaks showed the same B_12_ complementation ability than CN-Cbl standard (data not shown). Furthermore, higher corrinoid levels with DMB + CoCl_2_ addition compared with the basal condition (CoCl_2_ alone) were obtained. The corrinoid production in the presence of DMB was twofold and 2.2-fold greater for CRL 1098 and CRL 1001 strains, respectively, compared to the basal condition. The L-Thr + CoCl_2_ addition to the medium increased by 2.6-fold the corrinoid synthesis in *L. coryniformis* CRL 1001 respect to the basal condition (**Table 2**).

**Table 2:** Corrinoid compounds quantification by HPLC.

*L. reuteri* CRL 1098	Intermediaries	Corrinoid (μg/mL)^#^
	CoCl_2_	1.28 ± 0.26^∗^
	DMB + CoCl_2_	2.52 ± 0.20
*L. coryniformis* CRL 1001	Intermediaries	Corrinoid (μg/mL)
	CoCl_2_	1.34 ± 0.18
	DMB + CoCl_2_	2.98 ± 0.43
	L-Thr + CoCl_2_	3.45 ± 0.48

*The corrinoid concentration in CEs was calculated with a commercial CN-Cbl standard curve and the active peaks areas. ^#^Corrinoid concentration in CEs was calculated with a commercial CN-Cbl standard curve and the active peaks areas. ^∗^Standard deviation.*

**FIGURE 5:**
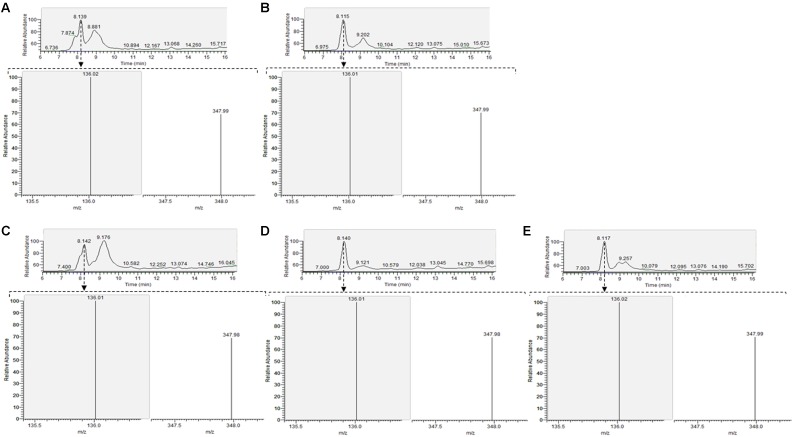
Liquid chromatography–electrospray ionization/tandem-mass spectrometry (LC/ESI–MS/MS) chromatograms of peaks with cobalamin activity. The transitions are shown together (SRMs, 672.5 m/z – > 136.0 m/z calculated for adenine y 672.5 m/z – > 348.0 m/z correspond to the lower ligand in which adenine is the aglycon attached to ribofuranose 3-phosphate). Total ion chromatogram (TIC) of the active peaks of *L. reuteri* CRL 1098 (**A**- CoCl_2_ **B**- DMB + CoCl_2_) and *L. coryniformis* CRL 1001 (**C**- CoCl_2_ **D**- DMB + CoCl_2_ **E**- L-Thr + CoCl_2_).

To characterize the corrinoid compounds, liquid chromatography–electrospray ionization/tandem mass spectrometry (LC/ESI–MS/MS) to the collected peaks was performed. Transitions were sought in the MS and MS/MS spectra of the peaks with cobalamin activity. The transitions 678.3 (m/z) [M + 2H+]++ to 358.7 (m/z) and 678.3 (m/z) [M + 2H+]++ to 146.9 (m/z) were examined for corrinoid compounds where DMB is the aglycon attached to ribofuranose 3-phosphate. For corrinoid compounds where adenine is the aglycon attached to ribofuranose 3-phosphate, transitions 672.5 (m/z) [M + 2H+]++ to 347.8 (m/z) and 672.5 (m/z) [M + 2H+]++ to 135.9 (m/z) were sought. For all active peaks analyzed, the MS spectra indicated that a double charged ion with an approximately m/z of 673 [M + 2H+]++ was prominent. The MS/MS spectrum showed that the dominant ions with a value approximate m/z 347.8 [M + 2H+]++ were attributable to Coα-[α-(7-adenyl)]-Coβ-cyanocobamide (Pseudo B12). In this compound type, adenine is the aglycon attached to ribofuranose-3-phosphate in the lower ligand (**Figure 5**).

### Expression of Key Genes of the Corrinoid Biosynthesis Pathway

Since the addition of biosynthetic intermediaries improved cobalamin production in both strains, the relative expression of key genes of the biosynthetic pathway in cells grown during 3 h (lag phase), 6 h (exponential phase), and 9 h (late-exponential phase) in B_12_ free medium in the presence of different intermediaries was tested by qPCR. The time of incubation (3, 6, and 9 h) in the B_12_ free medium with CoCl_2_ was the condition used as reference, to which the arbitrary value of 1 was assigned for both strains. Basal level of expression of key genes in the vitamin B_12_ free medium with different intermediaries was observed for both strains at all growth phases. The relative expression levels of the key genes with DMB and L-Thr addition remained unchanged in the strains under study after 3, 6, and 9 h of incubation with respect to the basal condition (data not shown). These data demonstrate that the key genes studied for *L. reuteri* CRL 1098 and *L. coryniformis* CRL 1001 may be regulated at a post-transcriptional or translational level.

## Discussion

We have previously reported that *L. reuteri* CRL 1098 supplementation efficiently correct the nutritional vitamin B12 deficiency in an *in vivo* model (Molina et al., 2008, 2009). In this work, cobalamin biosynthesis intermediaries were added to the vitamin B_12_ free medium to analyse their effect on the corrinoid production by two different *Lactobacillus* strains.

The common precursor of all tetrapyrrole molecules (cobalamin, heme, and chlorophyll) is ALA. The result of two condensed ALA molecules is PBG. Finally, four PBG molecules are polymerized and cyclized to form UIII, the last common intermediary (Kang et al., 2012). Mohammed et al. (2014) reported an increase in vitamin B_12_ production by *Bacillus* (*B*.) *megaterium* with addition of ALA to the culture medium (Mohammed et al., 2014). Different results were obtained for both *L. reuteri* CRL 1098 and *L. coryniformis* CRL 1001 strains, in which the decreased corrinoid compound production with ALA, PBG, and UIII addition may be due to a negative regulation by a classical feedback control (Robin et al., 1991).

Regarding CoCl_2_ and DMB addition, an increased cobalamin production is described for *Propionibacterium* and *Bacillus* strains (Mohammed et al., 2014; Wang et al., 2015). Similar results are reported for both *Lactobacillus* strains, CRL1001 and CRL1098, in the present study.

In the anaerobic pathway of the cobalamin biosynthesis, precorrin-2 is chelated with cobalt a reaction that is catalyzed by CbiK enzyme. Cobalt requirements for cobalamin-type corrinoids synthesis is proposed as screening criteria for detecting producer strains of *Propionibacterium* and *Lactobacillus* genera (Seidametova et al., 2004; Bhushan et al., 2016). In this work, we report that cobalt addition as chloride salt allowed the growth of both CRL 1098 and CRL 1001 *Lactobacillus* strain. Moreover, the cobalt addition also increased the corrinoid production in both strains compared with our previous data without cobalt addition (Taranto et al., 2003; Torres et al., 2016).

In another step of the biosynthesis, DMB is the nucleotide attached to the aminopropanol arm as lower ligand in the cobalamin molecule (Hazra et al., 2015). The cobalamin-type corrinoid compounds produced by CRL 1001 and CRL 1098 strains increased with DMB addition. Nevertheless, LC-MS results were different as expected since the molecules synthesized corresponded to pseudo-B_12_ despite the positive regulation on the synthesis. This compound, with adenine instead of DMB as lower ligand base is synthesized in most bacteria able to produce cobalamin-type corrinoid compounds via the anaerobic pathway (Taga and Walker, 2008). Hazra et al. (2013) proposed that CobT enzyme of *L. reuteri* CRL 1098 expressed in *E. coli* activates DMB rather than other molecules as base of the lower ligand (Hazra et al., 2013). According to our results, CRL 1098 strain is not able to use DMB instead of adenine for vitamin B_12_ synthesis. As previously published, *L. coryniformis* CRL 1001 does not have the *cobT* gene but the genes encoding CblS (a kinase enzyme) and CblT (a transporter protein) (Torres et al., 2016). Mattes and Escalante-Semerena (2017) observed that *cblST* genes of *Geobacillus* (*G.*) *kaustophilus* expressed in *S. enterica* Δ*cobT* that CblT transports DMB into the cell and CblS activate preferably ribazole over adenine (Mattes and Escalante-Semerena, 2017). Our results are not in agreement with this statement since pseudo-B_12_ is obtained, thus suggesting that CblS is unable to phosphorylate DMB despite entering the cell. Further studies are oncoming in both strains for a better understanding of the changes taking place in the lower ligand nucleotide of the molecule.

In a different way to the previously described, the L-Thr addition to the vitamin B_12_ free medium showed different results in *L. reuteri* CRL 1098 and *L. coryniformis* CRL 1001 strains. The increased production of cobalamin-type corrinoid compounds in CRL 1001 strain could be ascribed to the presence of *pduX* gene in the genome (Torres et al., 2016). The *pduX* encodes a protein kinase able to phosphorylate free L-Thr prior to the formation of lower ligand aminopropanol arm (Fan and Bobik, 2008). For this reason, we propose that L-Thr may exert some positive regulation in the synthesis of this type of compounds.

The study of the expression profile of key genes encoding proteins involved in cobalamin-type corrinoid compound synthesis showed no expression change despite the increased cobalamin production in both strains. Data obtained by different work groups showed that cobalamin synthesis is regulated by a riboswitch mechanism (Nahvi et al., 2004). Briefly, binding of cobalamin to the *cob* mRNA inhibits the initiation of translation and stabilizes the complex formed between them (Polaski et al., 2016). For these reasons, we suggest that the regulation is at the post-transcriptional or translational level. Our results are in agreement with previous reports since no changes in regulation of key synthesis genes of the mRNA transcription phase were evidenced (Nahvi et al., 2004).

In this study, we demonstrate that addition of key intermediaries to the vitamin B_12_ free medium increases the corrinoid production by both CRL1001 and CRL 1098 lactobacilli strains. In addition, the existence of two different cobalamin-type corrinoid compound biosynthetic pathways in two close phylogenetic strains was confirmed. The results obtained in this study would improve the production of these cobalamin-type corrinoid compounds by the food grade *L. coryniformis* CRL 1001 and *L. reuteri* CRL 1098 strains.

## Author Contributions

AT carried out biochemical and molecular genetic studies and participated in the drafting the manuscript. VV participated in physiological studies. GF participated in the discussion of the study. LS participated in the design, discussion, and coordination of this study and drafting the manuscript. MT carried out the coordination of this study and participated in the design, discussion, and drafting the manuscript. All authors read and approved the final manuscript.

## Conflict of Interest Statement

The authors declare that the research was conducted in the absence of any commercial or financial relationships that could be construed as a potential conflict of interest.
